# Dr. Claudius Henry Mastin

**Published:** 1898-11

**Authors:** 


Dr. Claudius Henry Mastin, of Mobile, Ala., died at his home in that city on Monday, October 3, 1898, aged 72 years. Though he had outlived the so-called allotted period of mankind he was always so vigorous and so much younger than his years, and withal, so rosy and cheerful, that his death came with a sudden shock to most of his friends in the North, and they were legion.

Dr. Mastin was born at Huntsville, Ala., June 4, 1826, received his academic degree at the University of Virginia twenty years later, and began the study of medicine in 1846 as a pupil of Dr. George B. Wood, at Philadelphia. He received his doctorate degree at the University of Pennsylvania in 1849, and in 1875 the honorary degree of LL. D. from the same source. Soon after graduating in medicine he passed several years in Europe, pursuing his studies in the great cities and returning in 1854 to establish himself in practice at Mobile. His early trend was in the direction of surgery and at once he attained reputation for skill in that branch. When the civil war came he entered the Confederate army, finally rising to the responsible position of medical director of the army corps commanded by Lieut. Gen. Leonidas Polk. He returned to Mobile at the end of the war and resumed his practice.

Dr. Mastin was an active member of nearly every national medical society of importance, as well as of his local and state organisations. He was an original member of the American Surgical Association; president in 1891. He was also a member of the American Genito-urinary Association, of the Southern Surgical and Gynecological Association, as well as of the American Medical Association. But he will best be known to posterity as the founder of the Congress of American Physicians and Surgeons, composed of the special societies, which meets in triennial sessions at Washington.

Dr. Mastin was a skilful physician and a keen diagnostician as well as a noted surgeon. His services as a consultant were demanded far and near both as physician and surgeon, and his loss will be keenly felt by a large number of physicians that weie accustomed to call for his services in this manner. He was an honorary member of many foreign as well as American medical societies, and among the latter of the American Association of Obstetricians and Gynecologists.

He is survived by a widow, two sons, two daughters and a brother —his sons and brother being themselves physicians.

Dr. Mastin was a vigorous writer and a frequent contributor to medical journals and magazines. He wrote a remarkable hand, steady, even, letters well formed, punctuation accurate and page after page fell from his pen without erasures, interlineations, or corrections in any form—it was perfect at first. Truly this remarkable man, whose fame was world-wide, will be mourned by people of both hemispheres.

